# Supplementary material for: The Highly Conservative Cysteine of Oncomodulin as a Feasible Redox Sensor
Source: Biomolecules. 2021 Jan 6;11(1):66. doi: 10.3390/biom11010066 (PMC7825312; doi:10.3390/biom11010066)
Supplement: Supplementary file 1 [file biomolecules-11-00066-s001.zip › Supplementary/Table S1.pdf]

Table S1. Values of the free energy of Ca^{2+}/Mg^{2+} binding to rWT/C18S Ocm, ΔG_i (i=1,2), calculated using the expression $\Delta G_i = - RT \cdot \ln(K_{ai} \cdot [H_2O])$ and the K_{ai} values listed in Table 4. The total free energy of metal binding is expressed as $\Delta G_\Sigma = \Delta G_1 + \Delta G_2$.

Ocm	***$\Delta G_1(Ca^{2+})$, kJ/mol***	***$\Delta G_2(Ca^{2+})$, kJ/mol***	***$\Delta G_\Sigma(Ca^{2+})$, kJ/mol***	***$\Delta G_1(Mg^{2+})$, kJ/mol***	***$\Delta G_2(Mg^{2+})$, kJ/mol***	***$\Delta G_\Sigma(Mg^{2+})$, kJ/mol***	$\frac{\Delta G_1(Mg^{2+})}{\Delta G_1(Ca^{2+})}$	$\frac{\Delta G_2(Mg^{2+})}{\Delta G_2(Ca^{2+})}$	$\frac{\Delta G_\Sigma(Mg^{2+})}{\Delta G_\Sigma(Ca^{2+})}$
rWT	-60.7±1.5	-51.9±1.6	-112.5±3.1	-38.7±0.2	-33.1±0.5	-71.8±0.7	0.637±0.015	0.640±0.010	0.640±0.010
C18S	-52.3±1.3	-53.9±1.2	-106.2±2.4	-36.2±0.2	-32.2±1.1	-68.5±1.3	0.693±0.015	0.597±0.006	0.643±0.006
