# Supplementary figures and images for: The Highly Conservative Cysteine of Oncomodulin as a Feasible Redox Sensor
Source: Biomolecules. 2021 Jan 6;11(1):66. doi: 10.3390/biom11010066 (PMC7825312; doi:10.3390/biom11010066)

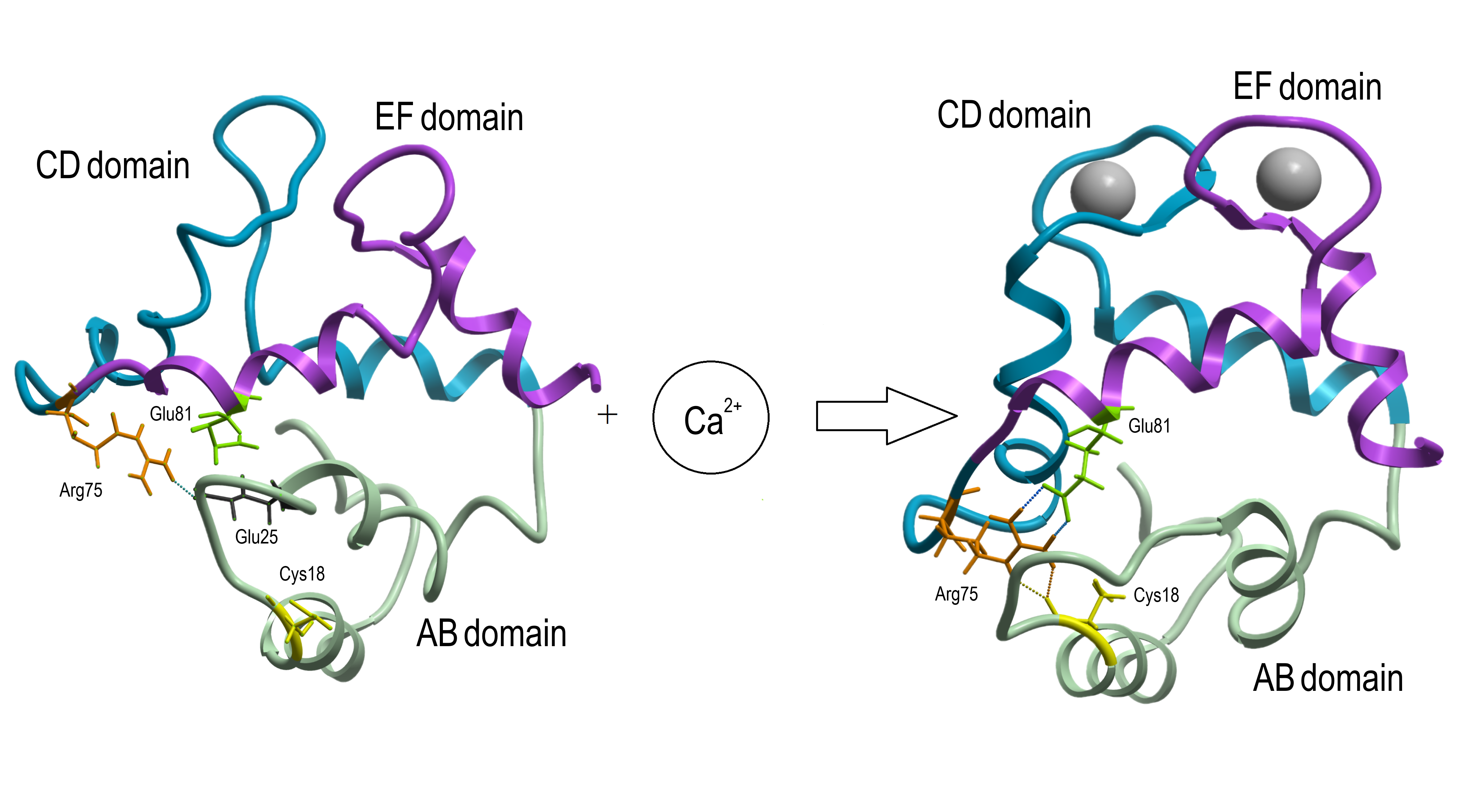

Supplement: Supplementary file 1 [file biomolecules-11-00066-s001.zip › Supplementary/Figure S1.tif]

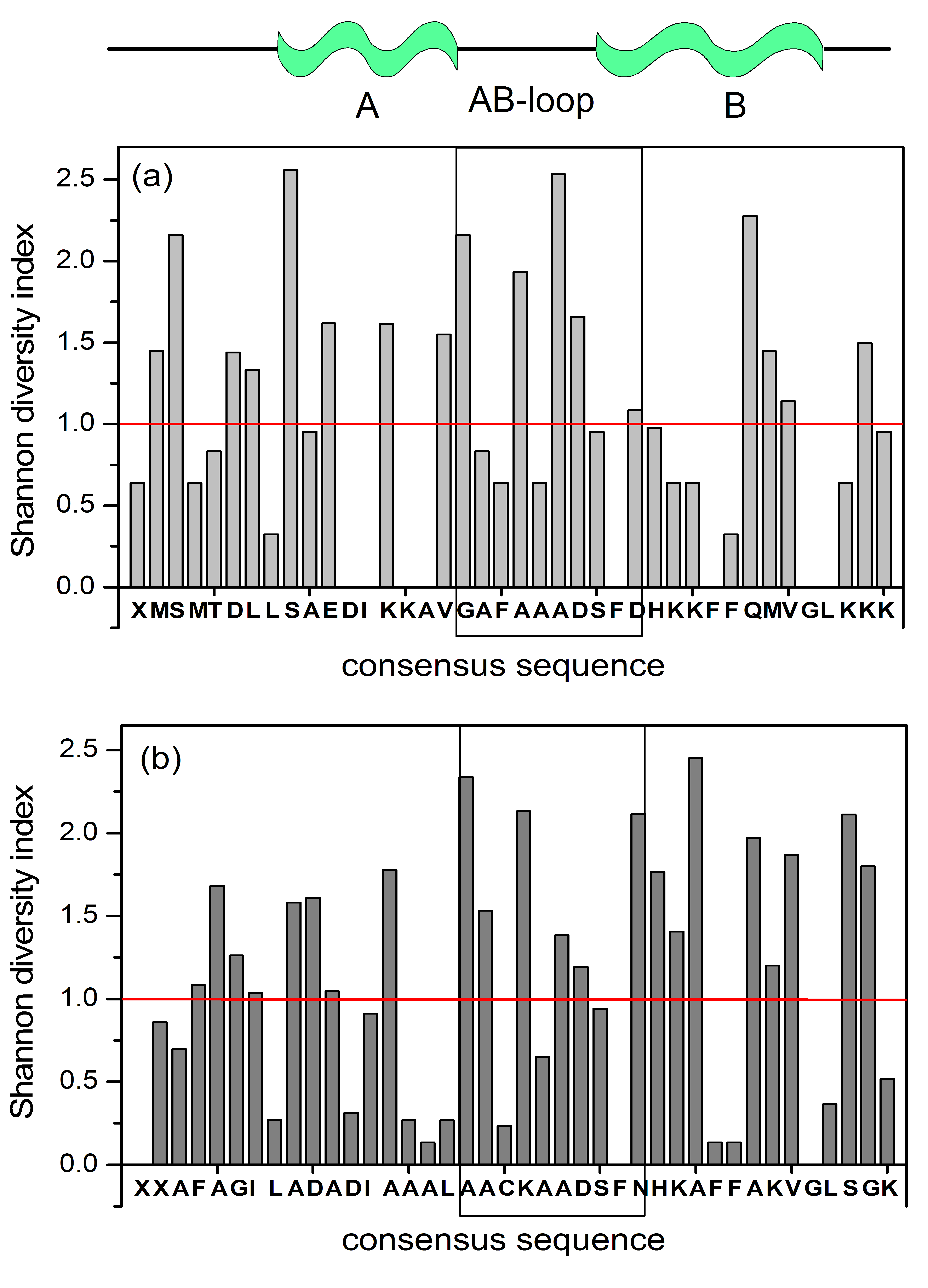

Supplement: Supplementary file 1 [file biomolecules-11-00066-s001.zip › Supplementary/Figure S4.tif]
